# Deprivation and exposure to public activities during the COVID-19 pandemic in England and Wales

**DOI:** 10.1136/jech-2021-217076

**Published:** 2021-10-12

**Authors:** Sarah Beale, Isobel Braithwaite, Annalan MD Navaratnam, Pia Hardelid, Alison Rodger, Anna Aryee, Thomas E Byrne, Erica Wing Lam Fong, Ellen Fragaszy, Cyril Geismar, Jana Kovar, Vincent Nguyen, Parth Patel, Madhumita Shrotri, Robert Aldridge, Andrew Hayward, Virus Watch Collaborative, Susan Michie

**Affiliations:** 1 Centre for Public Health Data Science, Institute of Health Informatics, University College London, London, UK; 2 Department of Epidemiology and Public Health, University College London, London, UK; 3 Centre for Paediatric Epidemiology and Biostatistics, UCL Institute of Child Health, London, UK; 4 Research Department of Infection and Population Health, Royal Free Campus, University College London, London, UK; 5 Department of Infectious Disease Epidemiology, London School of Hygiene and Tropical Medecine, London, UK

**Keywords:** COVID-19, deprivation, inequalities

## Abstract

**Background:**

Differential exposure to public activities may contribute to stark deprivation-related inequalities in SARS-CoV-2 infection and outcomes but has not been directly investigated. We set out to investigate whether participants in Virus Watch—a large community cohort study based in England and Wales—reported differential exposure to public activities and non-household contacts during the autumn–winter phase of the COVID-19 pandemic according to postcode-level socioeconomic deprivation.

**Methods:**

Participants (n=20 120–25 228 across surveys) reported their daily activities during 3 weekly periods in late November 2020, late December 2020 and mid-February 2021. Deprivation was quantified based on participants’ residential postcode using English or Welsh Index of Multiple Deprivation quintiles. We used Poisson mixed-effect models with robust standard errors to estimate the relationship between deprivation and risk of exposure to public activities during each survey period.

**Results:**

Relative to participants in the least deprived areas, participants in the most deprived areas exhibited elevated risk of exposure to vehicle sharing (adjusted risk ratio (aRR) range across time points: 1.73–8.52), public transport (aRR: 3.13–5.73), work or education outside of the household (aRR: 1.09–1.21), essential shops (aRR: 1.09–1.13) and non-household contacts (aRR: 1.15–1.19) across multiple survey periods.

**Conclusion:**

Differential exposure to essential public activities—such as attending workplaces and visiting essential shops—is likely to contribute to inequalities in infection risk and outcomes. Public health interventions to reduce exposure during essential activities and financial and practical support to enable low-paid workers to stay at home during periods of intense transmission may reduce COVID-related inequalities.

## Introduction

Socioeconomically deprived communities have been disproportionately impacted by the COVID-19 pandemic, experiencing higher rates of infection and mortality than less deprived communities as well as greater social and economic disruption.^1–4^

Public activities—such as working outside the home, visiting shops or using public transport—may promote the spread of SARS-CoV-2 through contact with potentially infectious individuals and through aerosol transmission.^5 6^ Consequently, ‘lockdown’ restrictions—including closure of or access limitations to non-essential shops and services, and recommendations to stay at home—have been a key pandemic response strategy worldwide.^7 8^ However, socioeconomic deprivation influences individuals’ ability to stay at home—for example, as a result of lower ability to work from home and greater reliance on public transport—as well as the level of contact with other people in the workplace. Consequently, differential exposure to public activities may contribute to higher rates of infections, and consequently hospitalisations and deaths from COVID-19, in deprived communities.^2 4^

Empirical investigation into the relationship between daily activities and risk of respiratory infection is limited. Public activities involving potentially poorly ventilated, high-footfall settings and/or close contact with others—for example, public transport, visiting cafes or restaurants, shops and supermarkets, venues such as cinemas, theatres and concerts, and social events such as parties—are associated with a risk of seasonal respiratory infection.^9^ While emerging findings from the COVID-19 pandemic show that measures limiting public mixing are associated with reduced SARS-CoV-2 transmission at the population level,^10–12^ data on the nature of individuals’ day-to-day activities are currently limited. Although differential exposure to public activities may influence socioeconomic inequalities in infection and mortality risk, data are lacking regarding how exposure to such activities varies in relation to sociodemographic characteristics, including deprivation.

Understanding how deprivation is associated with exposure to public activities is consequently important to ensure that public health and policy responses to COVID-19 and related inequalities are grounded in evidence. We set out to address this gap using data on public activities and non-household contacts collected through the Virus Watch cohort study.^13^

## Methods

### Survey procedure

We used data from three consecutive survey waves of the Virus Watch household cohort study.^13^ Households were recruited via post, social media, SMS messages and personalised letters disseminated by general practices. Eligibility criteria were residence in England or Wales, informed consent or assent for voluntary participation provided by all household members, internet access and an email address, at least one household member able to complete surveys in English, and household size between 1 and 6 household members (due to survey infrastructure limitations). Virus Watch study procedures are described in detail in the study protocol^13^ and include completion of monthly questionnaires into pandemic-relevant demographic, psychosocial/behavioural and health-related factors from which the current data were drawn.

Participants were prompted on 1 December 2020, 4 January 2021 and 17 February 2021 to complete an online questionnaire regarding their social activities and contacts during the preceding week. These survey weeks corresponded to key time points in terms of epidemic waves and/or government legislation regarding public activities in England and Wales. The first survey (covering 24 November 2020–1 December 2020) corresponded to the final week of the second English national lockdown,^14^ and the early stages of a sharp rise in COVID-19 cases and the emergence of the lineage B.1.1.7 variant. The second survey week (23 December 2020–27 December 2020) corresponded to the December holiday period, during which there was a notable variation in rules around social mixing across regions that was altered in the run-up to the holiday period due to sharp increases in COVID-19 cases in some regions. Indoor mixing with non-household members was not allowed in London, the South East or East of England, while indoor mixing on 25 December was allowed with a maximum of three other households across other English regions^15^ and with two other households in Wales.^16^ The third survey week (9 February 2021–16 February 2021) occurred during the third national lockdown for both England and Wales^16 17^—a period of deceleration in reported cases nationally.

Survey respondents reported the days they undertook a range of activities during each period (see the Outcomes section) and their number of non-household/support bubble close contacts (‘face-to-face contact with someone less than a metre away, even if a face-covering or mask was worn, or within 2 m for 15 min or more’).^18^ The wording of questions in the second and third surveys was edited to specifically refer to contact with non-household or support bubble members (vs non-household members) for clarity. Survey data were extracted on 25 February 2021.

### Exposure

The exposure of interest, deprivation, was measured at small local area level based on the Ministry for Housing and Local Government (in England) and Welsh Government (in Wales) Index of Multiple Deprivation (IMD) quintiles (1=most deprived, 5=least deprived). Participants provided household postcodes on study registration, which were used to derive IMD quintiles based on linkage with the May 2020 ONS Postcode Lookup file. Consequently, only survey respondents who provided a valid postcode at baseline were included in these analyses.

### Outcomes

The following activities were classified as binary outcomes of interest (yes/no during given period): driving or riding in a car/taxi with a non-household member, taking public transport (underground trains, overground trains, buses or trams), work/education (attending workplace or education settings outside the household), social/entertainment activities (defined as any of: attending the theatre, cinema, concert or sports event; eating in a restaurant, cafe or canteen; going to a bar, pub or club; and going to a party), going to essential shops, going to non-essential shops or personal care services, and close contact with one or more non-household/support bubble members. In the first and second surveys, exposure to car/taxis was asked as a single item; in the third survey, this item was disaggregated into separate car and taxi outcomes.

### Covariates

Age, sex and geographic region were considered relevant a priori potential confounders due to plausible relationships with both IMD and activities. Age and sex were derived from participants’ responses to demographic questions at study baseline. Age was classified as child (0–15 years), adult (16–64 years) and older adult (65+ years). Region was derived from linking participants’ postcode to ONS national region using the May 2020 National Statistics Postcode Lookup file. For the current analyses, regions were classified into the following three categories based on differing activity-related legislation and rates of SARS-CoV-2: London/South East/East of England (initial tier 4 regions), Wales and other regions.

### Statistical analyses

To assess the risk of reporting each activity by IMD quintile adjusted for age, sex, and region, we used Poisson mixed-effect models with robust standard errors^19^ using the mepoisson command in Stata V.16. We used Poisson mixed-effect models with robust standard errors—an established analytical approach to model relative risk for binary outcome variables^19^—to facilitate ease of interpretation compared with ORs, and modelled each activity outcome separately in order to investigate the frequency of specific activities by social group. All available data were entered into the models; data were complete for IMD and region and missing data were limited across time points for age group (range across time 0.29%–0.35%, n*=*59–84), sex (range: 1.06%–1.48%, n*=*217–310) and ethnicity (range across time: 1.24%–1.57%, n*=*249–330). The least deprived quintile (IMD 5) was used as the reference category. We included a random term to account for household-level clustering. We applied the Benjamini-Hochberg Procedure (false discovery rate: 0.05) to correct for multiple testing.

We performed a sensitivity analysis stratifying the relationship between IMD and attending work/education settings by age (child: <16 years vs adult: ≥16 years) to account for potential effect modification, as the impact of legislation around school openings may have had a more consistent effect on children across IMD quintiles than legislation around work/higher education for adults. As attendance of work/education settings is likely to influence non-household contacts, we also stratified the relationship between IMD and non-household contacts by age. For adults, we also performed a further sensitivity analysis for these outcomes controlling for the presence of children (<16 years) in the household. This was considered a potential confounder due to the negative association between maternal education (likely associated with small area level deprivation) and likelihood of having children,^20^ along with the likely influence of COVID-related school closures on the working patterns of parents and carers.

## Results

Table 1 reports the characteristics of the full Virus Watch cohort as of 25 February 2021 (n=46 539 individuals from 22 556 households) and of respondents to each activity survey who provided a valid postcode at baseline (n=20 120–25 228).

**Table 1:** Demographic features of survey respondents

		Virus Watch cohort	Survey 1 (24 November 2020–1 December 2020)	Survey 2 (23 December 2020–27 December 2020)	Survey 3 (9 February 2021–16 February 2021)
Total n		46 539	20 968	20 120	25 228
Age (years), % (n)*	0–15	12.35 (5729)	8.99 (1879)	7.74 (1553)	8.54 (2148)
	16–64	58.68 (27 220)	53.11 (11 098)	52.63 (10 559)	53.04 (13 337)
	65+	28.97 (13 437)	37.89 (7918)	39.62 (7949)	38.41 (9659)
	Missing	0.33 (153)	0.35 (73)	0.29 (59)	0.33 (84)
Sex - % (n)*	Female	55.43 (21 746)	54.89 (11 340)	55.14 (10 974)	54.58 (13 624)
	Male	44.57 (17 489)	45.11 (9318)	44.86 (8929)	45.42 (11 336)
	Missing	*15.69 (7304*)	1.48 (310)	1.08 (217)	1.06 (268)
Ethnicity - % (n)*	White British	85.13 (33 308)	89.36 (18 433)	89.42 (17 769)	88.13 (21 969)
	White Irish	1.47 (575)	1.36 (280)	1.32 (262)	1.40 (349)
	White Other	5.40 (2111)	4.41 (911)	4.36 (866)	4.71 (1174)
	South Asian	3.64 (1424)	1.64 (339)	1.77 (351)	2.31 (576)
	Other Asian	0.94 (369)	0.66 (137)	0.72 (143)	0.75 (186)
	Black	0.65 (253)	0.37 (77)	0.38 (76)	0.44 (111)
	Mixed	1.96 (769)	1.66 (343)	1.50 (298)	1.64 (408)
	Other ethnicity	0.52 (202)	0.39 (80)	0.34 (68)	0.42 (106)
	Prefer not to say	0.29 (115)	0.18 (38)	0.19 (38)	0.20 (50)
	Missing	15.93 (7413)	1.57 (330)	1.24 (249)	1.19 (299)
IMD, % (n)*	1 (most deprived)	5.83 (2397)	8.40 (1762)	8.10 (1630)	7.97 (2011)
	2	12.80 (5264)	15.20 (3188)	14.70 (2958)	14.98 (3780)
	3	20.13 (8282)	20.36 (4269)	20.33 (4090)	20.51 (5174)
	4	27.82 (11445)	26.14 (5482)	26.15 (5262)	26.28 (6629)
	5 (least deprived)	33.42 (13750)	29.89 (6267)	30.72 (6180)	30.26 (7634)
	Missing	11.61 (5401)	0.00 (0)	0.00 (0)	0.00 (0)
Region, % (n)*	East of England	21.71 (8932)	20.44 (4285)	22.66 (4560)	22.32 (5632)
	South East	18.96 (7799)	17.95 (3764)	18.72 (3767)	19.50 (4919)
	London	15.07 (6200)	12.29 (2577)	11.44 (2302)	13.00 (3280)
	North West	10.96 (4507)	11.87 (2488)	11.22 (2257)	11.07 (2793)
	East Midlands	8.52 (3505)	9.65 (2023)	9.21 (1953)	8.95 (2257)
	South West	7.05 (2900)	8.37 (1755)	7.92 (1593)	7.55 (1904)
	West Midlands	5.36 (2206)	6.07 (1272)	5.63 (1133)	5.48 (1383)
	North East	5.10 (2097)	5.38 (1128)	5.11 (1029)	4.96 (1252)
	Yorkshire and the Humber	4.82 (1983)	5.62 (1179)	5.17 (1040)	4.90 (1236)
	Wales	2.45 (1009)	2.47 (497)	2.42 (486)	2.27 (572)
	Missing	11.61 (5401)	0.00 (0)	0.00 (0)	0.00 (0)

*Percentages are expressed for total observed data for each category, while percentage missing is expressed for the full survey sample.

The proportion of participants reporting each activity by IMD quintile is presented in online supplemental tables 1–3.

10.1136/jech-2021-217076.supp1 Supplementary data

**Figure 1:**
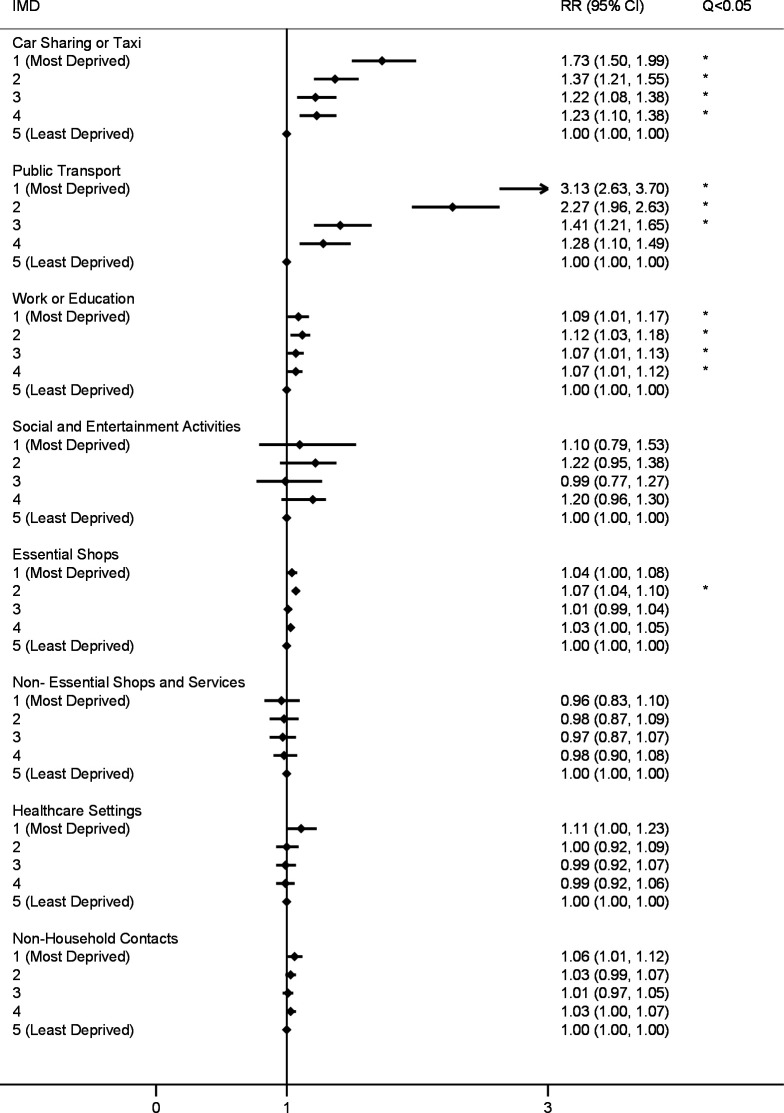
Risk ratios for public activities and non-household contacts by IMD quintile (24 November 2020–1 December 2020). Note: all models adjusted for participant age, sex and region of residence. Q <0.05 indicates that the Benjamini-Hochberg corrected p value (q value), which corrects for multiple comparisons, falls below <0.05. RR, risk ratio. IMD, Index of Multiple Deprivation.

**Figure 2:**
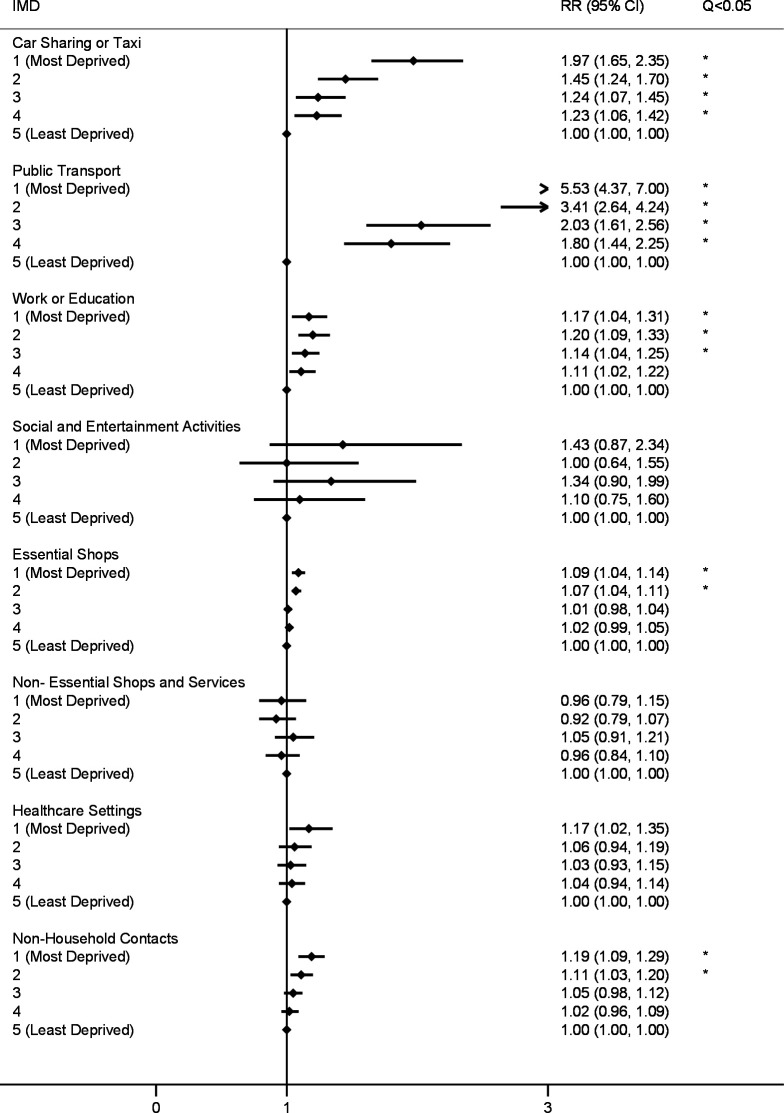
Risk ratios for public activities and non-household contacts by IMD quintile (23 December 2020–27 December 2020). Note: all models adjusted for participant age, sex and region of residence. Q <0.05 indicates that the Benjamini-Hochberg corrected p value (q value), which corrects for multiple comparisons, falls below <0.05. RR, risk ratio. IMD, Index of Multiple Deprivation.

**Figure 3:**
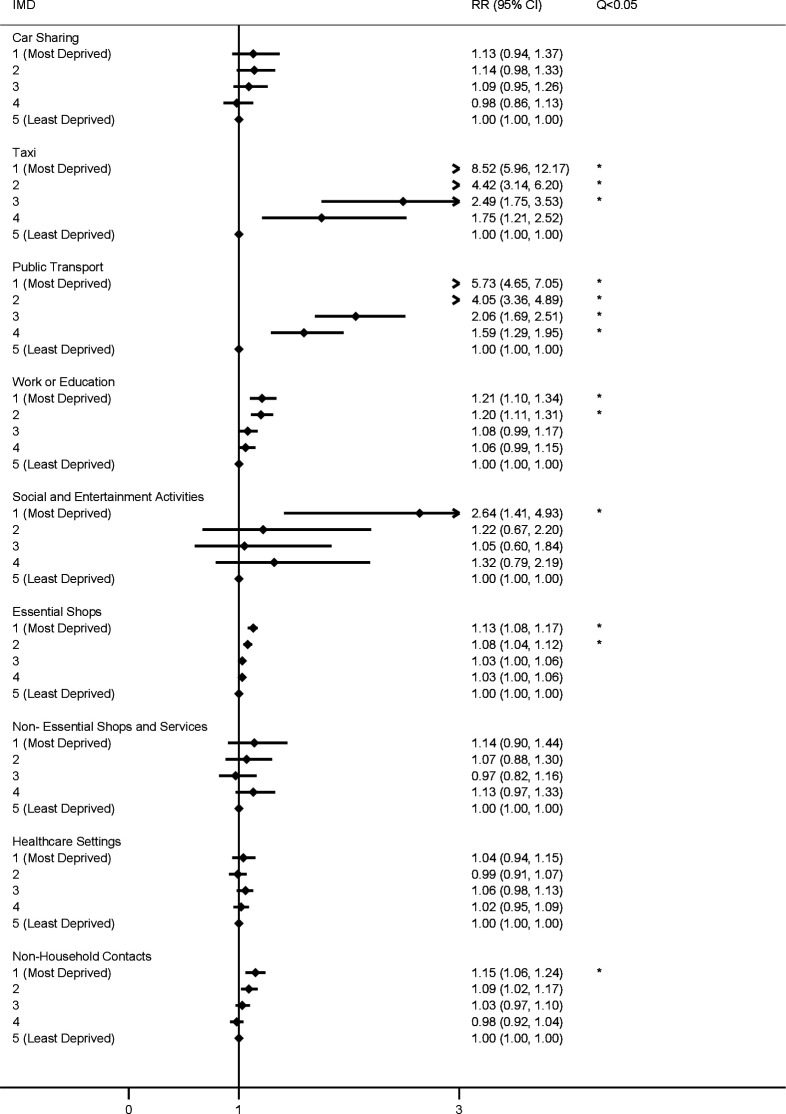
Risk ratios for public activities and non-household contacts by IMD quintile (09 February 21–16 Febrry 21). Note: all models adjusted for participant age, sex and region of residence. Q <0.05 indicates that the Benjamini-Hochberg corrected p value (q value), which corrects for multiple comparisons, falls below <0.05. RR, risk ratio. IMD, Index of Multiple Deprivation.

Poisson mixed models for the first (24 November 2020–1 December 2020; figure 1) and second (23 December 2020–27 December 2020; figure 2) survey periods indicated that—compared with the least deprived group—participants in all other IMD quintiles had elevated risk following multiple comparison correction for vehicle sharing (adjusted risk ratio (aRR) range, respectively: 1.22 (1.08–1.38)–1.73 (1.50–1.99) and 1.24 (1.07–1.45)–1.97 (1.65–2.35)). Car sharing versus taxi use was disaggregated in the third survey period (9 February 2021–16 February 2021; figure 3), and IMD 1–3 had elevated risk of taxi use compared with the least deprived group (aRR range: 2.49 (1.75–3.53)–8.52 (4.65–7.05)). No difference was found by IMD for car sharing, which was less-commonly reported overall (online supplemental table 2).

In the first survey, participants in all deprivation groups demonstrated elevated risk of exposure to workplace or education settings outside the home compared with the least deprived quintile (aRR range: 1.07 (1.01–1.12)–1.12 (1.03–1.18)). Similar findings regarding elevated risk of exposure to workplace and education settings emerged in the second survey for IMD quintiles 1–3 (aRR range: 1.14 (1.04–1.25)–1.20 (1.09–1.33)) and IMD 1–2 in the third survey (aRR, respectively: 1.21 (1.10–1.34) and 1.20 (1.11–1.31)). Across survey periods, these findings applied only to adults after stratification by age (online supplemental table 4) and remained following adjustment for the presence of children in the household (online supplemental table 6).

Regarding exposure to public transport, participants in IMD quintiles 1–3 had an elevated risk of exposure in the first survey (aRR range: 1.41 (1.21–1.65)–3.13 (2.63–3.70)), and participants in all investigated IMD quintiles had elevated risk in the second (aRR range: 1.80 (1.44–2.45)–5.53 (4.37–7.00)) and third (aRR range: 1.59 (1.29–1.95)–5.73 (4.65–7.05)) surveys compared with the least deprived group. Across all three surveys, CIs for IMD quintiles 1–2 indicated elevated risk for public transport compared with all other quintiles (figures 1–3).

Risk of exposure to essential shops was elevated for IMD quintile 2 (aRR: 1.07 (1.04–1.10)) in the first survey, and for IMD quintiles 1 and 2 in the second (aRR, respectively: 1.09 (1.04–1.14) and 1.07 (1.04–1.11)) and third (aRR, respectively: 1.13 (1.08–1.17) and 1.08 (1.04–1.12)) surveys.

No difference in risk of exposure to non-household contacts emerged in the main analyses in the first survey, while IMD 1 and 2 (aRR, respectively: 1.19 (1.09–1.29) and 1.11 (1.03–1.20)) and IMD 1 demonstrated elevated risk (aRR: 1.15 (1.06–1.24)) in surveys 2 and 3, respectively. Across survey periods, age-stratified analysis (online supplemental table 5) indicated elevated exposure to non-household contacts for adults in IMD 1 and 2, which persisted after controlling for the presence of children in the household (online supplemental table 6).

For the first time across measured time points, participants in IMD 1 in the third survey were also more likely to report social and entertainment activities (aRR: 2.64 (1.41–4.93)). No differences emerged by IMD for visiting non-essential shops or healthcare settings for any survey period.

## Discussion

Our findings suggest that differences in essential daily activities—in particular, using public transport, attending work/education settings outside the home and visiting essential shops—likely contribute to elevated risk of COVID-19 infection and mortality in deprived communities. Patterns of differential exposure to essential activities were consistent across the three survey periods. Adults—but not children—consistently demonstrated differential exposure to workplace/education settings and non-household contacts, likely reflecting the effects of legislation around school openings for children. We found limited evidence of deprivation-related differences for activities more reflective of individual choice, with no differences in visiting non-essential shops or services and elevated risk of social and entertainment activities in the most deprived group at one time point only (February 2021).

This is the first study to investigate the relationship between deprivation and exposure to specific daily activities during the COVID-19 pandemic. The Virus Watch cohort comprises households from across England and Wales with considerable diversity in age, ethnicity and area-level deprivation. The Virus Watch recruitment strategy aimed to recruit households from a diverse range of communities. However, response to Virus Watch surveys was voluntary and survey respondents were not demographically representative of the population, with a lower proportion of the survey samples drawn from the most deprived communities (~8%) compared with the least deprived (~30%), potentially affecting generalisability of responses from the most deprived groups as well as statistical power. Where possible, replication within a population-representative sample is recommended. Self-reported activities may have been affected by recall and social desirability biases. Social desirability bias is an important limitation of self-reported activity measures, particularly as some activities included in the surveys were illegal during the survey periods (eg, attending a party). However, unlike objective movement tracking, self-report surveys allow detail around the specific nature of the activity and bias may have been minimised by online survey delivery. A further limitation was the inability to quantify infection risk associated with each activity due to insufficient data. This will be the focus of future work when additional virological and serological outcome data are available. Quantifying the number and intensity of contacts per setting and any risk mitigation strategies were also beyond the scope of these surveys. Despite relevant age-stratified analysis, we were not able to directly distinguish between workplace and education settings in the current study. IMD is also an area-level measure and may not always reflect individuals’ socioeconomic position. Further investigation into the influence of individual-level indicators of socioeconomic position, including occupation and education, is warranted, as is investigation into the interrelationship between deprivation, ethnicity and infection risk. Investigating workplace attendance by occupation is also an important area for further investigation given differential exposure to workplace/education settings for adults.

In the UK, lockdown and social distancing measures appear to have reduced contacts and mobility in public locations compared with pre-pandemic levels^21–23^; however, the current findings suggest that deprivation-related differences in exposure to essential public activities are consistently present during periods of stringent regulations. These findings are consistent with a US-based study^24^ that constructed granular spatio-temporal mobility networks based on mobile phone data for 98 million people across ~57 000 neighbourhoods. Integrating these networks within a metapopulation susceptible-exposed-infectious-recovered model allowed an accurate fit to observed case trajectories and predicted ethnicity-related and deprivation-related risk gradients. This study found that disadvantaged groups’ mobility did not reduce as sharply as people living in majority white and higher-income areas, and that the locations that they visited were typically smaller and more crowded.

Interpretation of activity and contact patterns reported in the current study should take into account changes in the wider context at these times. The lockdown restrictions that were in place during the November and February surveys were similar in England; key differences being the closure of primary and secondary schools and increased fines in January and February.^14 17^ The English policy context surrounding the December survey differed substantially: different ‘tier’-based restrictions were in place across England, and rules for contact on 25 December (Christmas Day) were relaxed only in some regions. No indoor mixing with non-household members was allowed in London, the South East or East of England; other English regions were allowed meeting a maximum of three households.^15^ In Wales, the late November survey fell 2 weeks after the 17-day national ‘firebreak’ lockdown ended, and a further Welsh lockdown started on 20 December,^16^ with rules relaxed to allow mixing with up to two other households on 25 December only.^16^ In February, Wales was again under lockdown. Differential exposure to essential activities and non-household contacts was consistently observed across survey periods. Consequently, deprivation-related inequalities in infection and mortality risk driven by essential public activities may persist or become exacerbated during periods of stringent lockdown restrictions. Measuring differential activities during periods of less intensive or no restrictions on social mixing is consequently warranted.

The current findings suggest that interventions to reduce SARS-CoV-2 exposure on public transport, in essential shops, and in workplace and education settings where in-person attendance is required (eg, through measures to improve ventilation, face covering use where appropriate, testing and isolation) may reduce inequalities in infection risk. Deprivation-related differences in exposure to these essential activities are likely to reflect structural factors that constrain individual choice, such as car ownership, ability to work from home and disposable income. Providing greater financial and practical support to facilitate increased uptake of testing and adherence to isolation when required may also prevent mortality and reduce inequalities.^25^ Interventions and public health communications targeting activities that do not consistently differ between social groups over time, such as fines for attending social activities, conversely appear unlikely to reduce COVID-related inequalities.

**What is already known on this subject**

- People from socioeconomically deprived communities have experienced disproportionate risk of infection and mortality during the COVID-19 pandemic. Evidence from non-COVID respiratory illnesses indicates that public activities—such as using public transport, visiting large shops and supermarkets, and social activities—are associated with becoming ill. Deprivation is likely to influence individuals’ ability to limit public exposure, and consequently infection risk, during the COVID-19 pandemic; however, empirical investigation into activity patterns by sociodemographic characteristics is lacking.

**What this study adds**

- People living in the most deprived regions of England and Wales had elevated risk of exposure to vehicle sharing, public transport, work or education settings, essential shops and non-household contacts across three survey periods during the autumn/winter wave of the COVID-19 pandemic. Consistently elevated exposure to these essential public activities is likely to contribute to differential infection risk in deprived groups that persists during periods of stringent restrictions on social mixing. Measures to mitigate infection risk during essential activities, as well as financial and practical support to limit public exposure during periods of intense transmission are indicated to address deprivation-related inequalities during the current pandemic.

## Data Availability

No data are available. We aim to share aggregate data from this project on our website and via a "Findings so far" section on our website - *https://ucl-virus-watch.net/*. We will also be sharing individual record level data with personal identifiers removed on a research data sharing service such as the Office of National Statistics Secure Research Service. In sharing the data we will work within the principles set out in the UKRI Guidance on best practice in the management of research data. Access to use of the data whilst research is being conducted will be managed by the Chief Investigators (ACH and RWA) in accordance with the principles set out in the UKRI guidance on best practice in the management of research data. It is the intention that the data arising from this research will initially be collected, cleaned and validated by the UCL research team and once this has been completed will be shared for wider use. We aim to make subsets of the data more rapidly available both on our study website and via the public facing dashboard during the ongoing phase of data collection. In line with Principle 5 of the UKRI guidance on best practice in the management of research data, we plan to release data in batches as they become available or as updated results are published. Individual record data linked using NHS Digital will not be shared, only aggregated results. HES and mortality data may be obtained from a third party and are not publicly available. These data are owned by a third party and can be accessed by researchers applying to the Health and Social Care Information Centre for England. We will put analysis code on publicly available repositories to enable their reuse.
