# Supplementary material for: Deprivation and exposure to public activities during the COVID-19 pandemic in England and Wales
Source: J Epidemiol Community Health. 2021 Oct 12;76(4):319–26. doi: 10.1136/jech-2021-217076 (PMC8520599; doi:10.1136/jech-2021-217076)
Supplement: Supplementary data [file jech-2021-217076supp001.pdf]

Supplementary Material

Supplementary Table 1.

Proportion of participants reporting each activity and total number of respondents by deprivation quintile (24 Nov 20 – 01 Dec 20)

IMD Quintile	Car Sharing or Taxi		Public Transport		Work or Education		Social/ Entertainment		Essential Shops		Non-Essential Shops/Services		Healthcare Setting		Non-Household Contacts	
	Yes % (*n*)	total *n*	Yes % (*n*)	total *n*	Yes % (*n*)	total *n*	Yes % (*n*)	total *n*	Yes % (*n*)	total *n*	Yes % (*n*)	total *n*	Yes % (*n*)	total *n*	Yes % (*n*)	total *n*
**1 (Most Deprived)**	19.52 (344)	1762	16.52 (291)	1762	38.57 (680)	1763	4.20 (74)	1762	72.04 (1270)	1763	17.58 (310)	1763	24.52 (432)	1762	65.06 (1145)	1760
**2**	15.63 (498)	3188	14.20 (450)	3186	36.59 (1166)	3187	4.84 (154)	3184	74.91 (2389)	3189	17.86 (569)	3185	23.05 (734)	3184	62.77 (2003)	3191
**3**	14.34 (612)	4269	9.49 (405)	4267	33.52 (1431)	4269	3.89 (166)	4267	72.52 (3096)	4269	17.71 (756)	4268	23.63 (1008)	4266	60.97 (2599)	4263
**4**	14.30 (784)	5482	7.89 (432)	5478	32.88 (1801)	5478	4.56 (250)	5477	73.64 (4034)	5478	18.39 (1007)	5476	23.52 (1289)	5480	61.88 (3391)	5480
**5 (Least Deprived)**	11.54 (723)	6267	6.27 (393)	6266	31.16 (1952)	6268	4.12 (258)	6268	71.68 (4493)	6268	18.69 (1171)	6266	23.92 (1499)	6268	59.85 (3748)	6262
**Total**	14.12 (2961)	20968	9.40 (1971)	20959	33.54 (7031)	20965	4.30 (902)	20958	72.89 (15282)	20967	18.19 (3813)	20958	23.67 (4962)	20960	61.49 (12886)	20956

Note: IMD = Indices of Multiple Deprivation; total respondents for each question reported separately due to varying frequencies across questions

Supplementary Table 2.

Proportion of participants reporting each activity and total number of respondents by deprivation quintile (23 Dec 20 – 27 Dec 20)

IMD Quintile	total *n*	Car Sharing or Taxi Yes % (*n*)	Public Transport Yes % (*n*)	Work or Education Yes % (*n*)	Social/Entertainment Yes % (*n*)	Essential Shops Yes % (*n*)	Non-Essential Shops and Services Yes % (*n*)	Healthcare Setting Yes % (*n*)	Non-Household Contacts Yes % (*n*)
**1 (Most Deprived)**	1630	16.20 (264)	12.33 (201)	22.39 (365)	2.33 (38)	67.85 (1106)	11.66 (190)	16.26 (265)	40.92 (667)
**2**	2958	12.07 (357)	8.92 (264)	21.20 (627)	1.52 (45)	67.04 (1983)	10.18 (301)	14.91 (441)	37.19 (1100)
**3**	4090	10.76 (440)	5.79 (237)	18.90 (773)	2.30 (94)	63.28 (2588)	10.73 (439)	14.91 (610)	34.38 (1406)
**4**	5262	10.49 (552)	5.02 (264)	18.26 (961)	1.94 (102)	64.61 (3400)	10.70 (563)	15.18 (799)	33.69 (1773)
**5 (Least Deprived)**	6180	8.72 (539)	2.83 (175)	16.49 (1019)	1.68 (104)	63.40 (3918)	11.02 (681)	14.63 (904)	33.03 (2041)
**Total**	20120	10.70 (2152)	5.67 (1141)	18.61 (3745)	1.90 (383)	64.59 (12995)	10.81 (2174)	15.00 (3019)	34.73 (6987)

Note: IMD = Indices of Multiple Deprivation

Supplementary Table 3.

Proportion of participants reporting each activity and total number of respondents by deprivation quintile (09 Feb 21 – 16 Feb 21)

**IMD Quintile**		**Car Sharing**	**Taxi**	**Public Transport**	**Work or Education**	**Social/Entertainment**	**Essential Shops**	**Non-Essential Shops and Services**	**Healthcare Setting**	**Non-Household Contacts**
	total *n*	Yes % (*n*)	Yes % (*n*)	Yes % (*n*)	Yes % (*n*)	Yes % (*n*)	Yes % (*n*)	Yes % (*n*)	Yes % (*n*)	Yes % (*n*)
**1 (Most Deprived)**	2011	9.70 (195)	5.72 (115)	11.93 (240)	25.21 (507)	0.99 (20)	66.53 (1338)	6.46 (130)	22.18 (446)	40.13 (807)
**2**	3780	9.44 (357)	3.33 (126)	10.16 (384)	23.60 (892)	0.48 (18)	63.97 (2418)	5.95 (225)	21.61 (817)	37.83 (1430)
**3**	5174	8.95 (463)	2.03 (105)	5.68 (294)	20.00 (1035)	0.43 (22)	61.77 (3196)	5.51 (285)	24.08 (1246)	35.52 (1838)
**4**	6629	8.33 (552)	1.48 (98)	4.22 (280)	19.79 (1312)	0.69 (46)	62.50 (4143)	6.59 (437)	23.50 (1558)	33.50 (2221)
**5 (Least Deprived)**	7634	8.45 (645)	0.83 (63)	2.67 (204)	18.77 (1433)	0.45 (34)	60.61 (4627)	5.86 (447)	23.08 (1762)	34.35 (2622)
**Total**	25228	8.77 (2212)	2.01 (507)	5.56 (1402)	20.53 (5179)	0.55 (140)	62.32 (15722)	6.04 (1524)	23.11 (5829)	35.35 (8918)

Note: IMD = Indices of Multiple Deprivation

Supplementary Table 4.

Risk Ratios for Work and Education by IMD Quintile for Children (<16 years) versus Adults (≥16 years)

**IMD Quintile**	**24 Nov 20 – 01 Dec 20**		**23 Dec 20 – 27 Dec 20**		**09 Feb 21 – 16 Feb 21**	
	**Child**	**Adult**	**Child**	**Adult**	**Child**	**Adult**
**1**	0.96 [0.89,1.04]	***1.30* [1.18,1.43]***	0.53 [0.31,0.91]	***1.41* [1.25,1.59]***	0.90 [0.62,1.30]	***1.39* [1.26,1.55]***
**2**	0.94 [0.87,1.01]	***1.28* [1.18,1.39]***	0.90 [0.59,1.36]	***1.31* [1.18,1.45]***	0.71 [0.50,1.00]	***1.33* [1.22,1.45]***
**3**	0.98 [0.92,1.04]	1.13 [1.04,1.22]	0.67 [0.44,1.01]	***1.21* [1.09,1.34]***	0.66 [0.48,0.92]	1.13 [1.04,1.23]
**4**	1.03 [0.98,1.08]	1.09 [1.01,1.17]	0.87 [0.61,1.25]	1.12 [1.02,1.23]	0.86 [0.66,1.14]	1.08 [1.00,1.17]
**5**	REF	REF	REF	REF	REF	REF

Note: IMD = Indices of Multiple Deprivation (1 = most deprived; 5 = least deprived); 95% confidence intervals in parentheses
**** Bonferroni-Hochberg adjusted p<0.05***

Supplementary Table 5.

Risk Ratios for Non-Household Contacts by IMD Quintile for Children versus Adults

IMD Quintile	24 Nov 20 – 01 Dec 20		23 Dec 20 – 27 Dec 20		09 Feb 21 – 16 Feb 21	
	Child	Adult	Child	Adult	Child	Adult
**1**	0.92 [0.85,1.01]	***1.11* [1.06,1.18]***	0.76 [0.54,1.08]	***1.27* [1.17,1.38]***	0.93 [0.72,1.21]	***1.20* [1.11,1.29]***
**2**	0.92 [0.85,1.00]	***1.07* [1.02,1.12]***	0.97 [0.74,1.27]	***1.14* [1.06,1.23]***	0.91 [0.72,1.16]	***1.12* [1.05,1.20]***
**3**	***0.91* [0.84,0.97]***	1.03 [0.99,1.08]	0.70 [0.53,0.93]	1.08 [1.01,1.16]	0.73 [0.57,0.93]	1.06 [1.00,1.13]
**4**	0.99 [0.93,1.04]	1.04 [1.00,1.09]	0.75 [0.58,1.96]	1.04 [0.98,1.11]	0.96 [0.79,1.17]	0.98 [0.93,1.04]
**5**	REF	REF	REF	REF	REF	REF

Note: IMD = Indices of Multiple Deprivation (1 = most deprived; 5 = least deprived); 95% confidence intervals in parentheses
**** Bonferroni-Hochberg adjusted p<0.05***

Supplementary Table 6.

Risk Ratios for Exposure to Work and Education Settings and Non-Household Contacts by IMD Quintile for Adults - Controlling for Presence of Child(ren) in the Household

IMD Quintile	24 Nov 20 – 01 Dec 20		23 Dec 20 – 27 Dec 20		09 Feb 21 – 16 Feb 21	
	Work or Education	Non-Household Contacts	Work or Education	Non-Household Contacts	Work or Education	Non-Household Contacts
1	***1.28 [1.17, 1.41]****	***1.11 [1.05, 1.17]****	***1.38 [1.23, 1.56]****	***1.26 [1.16, 1.38]****	***1.38 [1.25, 1.53]****	***1.20 [1.11, 1.29]****
2	***1.29 [1.19, 1.39]****	***1.07 [1.02, 1.12]****	***1.31 [1.18, 1.45]****	***1.14 [1.06, 1.23]****	***1.34 [1.23, 1.46]****	***1.12 [1.05, 1.20]****
3	***1.14 [1.06, 1.23]****	1.03 [0.99, 1.08]	***1.22 [1.10, 1.34]****	***1.08 [1.01, 1.16]****	***1.14 [1.05, 1.24]****	***1.06 [1.00, 1.13]****
4	1.09 [1.02, 1.17]	1.05 [1.00, 1.09]	1.13 [1.03,1.24]	1.04 [0.98, 1.11]	1.09 [1.01, 1.18]	0.98 [0.93, 1.04]
5	REF	REF	REF	REF	REF	REF

Note: IMD = Indices of Multiple Deprivation (1 = most deprived; 5 = least deprived); 95% confidence intervals in parentheses
**** Bonferroni-Hochberg adjusted p<0.05***
